# Mid-Season Estimates of Influenza Vaccine Effectiveness against Influenza A(H3N2) Hospitalization in the Elderly in Quebec, Canada, January 2015

**DOI:** 10.1371/journal.pone.0132195

**Published:** 2015-07-22

**Authors:** Rodica Gilca, Danuta M. Skowronski, Monique Douville-Fradet, Rachid Amini, Nicole Boulianne, Isabelle Rouleau, Christine Martineau, Hugues Charest, Gaston De Serres

**Affiliations:** 1 Biological, Environmental and Occupational Risks, Institut National de Santé Publique du Québec, Quebec, QC, Canada; 2 CHU de Quebec, Quebec, QC, Canada; 3 Laval University, Quebec, QC, Canada; 4 Influenza & Emerging Respiratory Pathogens, British Columbia Centre for Disease Control, Vancouver, British Columbia, Canada; 5 Bureau de surveillance et de vigie, Ministère de la Santé et des Services sociaux du Québec, Quebec, QC, Canada; 6 Laboratoire de santé publique du Québec, Institut National de Santé Publique du Québec, Montreal, Canada; National Institutes of Health, UNITED STATES

## Abstract

**Background:**

The 2014/15 influenza season in Canada was characterized by an early epidemic due to vaccine-mismatched influenza A(H3N2) viruses, disproportionately affecting elderly individuals ≥65-years-old. We assessed vaccine effectiveness (VE) against A(H3N2) hospitalization among elderly individuals during the peak weeks of the 2014/15 epidemic in Quebec, Canada.

**Methods:**

Nasal specimens and clinical/epidemiological data were collected within 7 days of illness onset from elderly patients admitted with respiratory symptoms to one of four participating hospitals between November 30, 2014 and January 13, 2015. Cases tested RT-PCR positive for influenza A(H3N2) and controls tested negative for any influenza. VE was assessed by test-negative case-control design.

**Results:**

There were 314 participants including 186 cases (62% vaccinated) and 128 controls (59% vaccinated) included in primary VE analysis. Median age was 81.5 years, two-thirds were admitted from the community and 91% had underlying comorbidity. Crude VE against A(H3N2) hospitalization was -17% (95%CI: -86% to 26%), decreasing to -23% (95%CI: -99 to 23%) with adjustment for age and comorbidity, and to -39% (95%CI: -142 to 20%) with additional adjustment for specimen collection interval, calendar time, type of residence and hospital. In sensitivity analyses, VE estimates were improved toward the null with restriction to participants admitted from the community (-2%; 95%CI: -105 to 49%) or with specimen collection ≤4 days since illness onset (- 8%; 95%CI: -104 to 43%) but further from the null with restriction to participants with comorbidity (-51%; 95%CI: -169 to 15%).

**Conclusion:**

The 2014/15 mismatched influenza vaccine provided elderly patients with no cross-protection against hospitalization with the A(H3N2) epidemic strain, reinforcing the need for adjunct protective measures among high-risk individuals and improved vaccine options.

## Introduction

The 2014/15 influenza season in Quebec, as elsewhere in Canada, was characterized by an early and intense influenza epidemic due almost exclusively to antigenically-drifted and vaccine-mismatched A(H3N2) viruses [1, 2]. As expected with influenza seasons dominated by A(H3N2) subtype activity, the elderly ≥65 years-old were disproportionately affected by excess hospitalizations and deaths [3, 4]. By mid-season in some jurisdictions, the number of long-term care facility (LTCF) outbreaks in 2014/15 exceeded even the full-season tallies of recent prior seasons, including those also distinguished by dominant, vaccine-mismatched A(H3N2) activity, such as 2012/13 [2, 5, 6].

In response to surveillance signals suggesting suboptimal vaccine performance, several mid-season analyses assessed effectiveness of the 2014/15 influenza vaccine against the A(H3N2) epidemic strain. Canada’s Sentinel Physician Surveillance Network (SPSN) measured vaccine effectiveness (VE) against medically-attended laboratory-confirmed outpatient A(H3N2) illness of -8% (95%CI:-50–23%) overall and 2% (95%CI:-49–36%) in non-elderly (<65-year-old) adults, indicating little or no vaccine protection even among individuals capable of mounting an effective immune response [2]. The Canadian Immunization Research Network (CIRN) assessed VE against influenza A(H3N2)-related hospitalization, reporting estimates partially adjusted for age and comorbidity of 8% (95%CI:-102–58%) in non-elderly adults, substantially lower in elderly adults at -33% (95%CI:-104–13%) [7].

Although Canadian mid-season inpatient and outpatient VE findings for the 2014/15 season have been consistent with null vaccine effects (statistically non-significant and spanning zero) in both age groups, the CIRN finding of a lower and negative point estimate of VE against A(H3N2) hospitalization in the elderly, more closely broaching statistical significance, warrants further clarification. Here we assess VE against A(H3N2) hospitalization in the elderly during the peak epidemic weeks of the 2014/15 season in Quebec, Canada.

## Methods

### Study design and participants

This study was conducted under the surveillance mandate of the Quebec Ministry of Health without requirement for Institutional review board approval. As part of routine patient care, all patients admitted to hospitals participating in the project with respiratory symptoms are assessed for influenza by per-nasal specimen collection at local laboratory. All patients admitted ≥24 hours at one of these sentinel hospitals with cough, sore throat, or fever/feverishness of unknown etiology were invited by a research nurse to participate in the study. Authors themselves did not have direct contact with patients or access to patient identifying information and no additional samples were collected for the purposes of research. Verbal consent was elicited from the patient and/or guardian to test the specimen for influenza and other respiratory viruses at the provincial public health laboratory and to record demographic and clinical information such as influenza vaccination, and date of illness onset on standardized questionnaires. Patient charts were also reviewed at discharge to collect information on clinical progress. Verbal consent was documented on the questionnaire. Capacity to consent was determined by the nurse; the number of patients unable or refusing to give consent was recorded weekly on recruitment files and qualified as exclusions.

**Fig 1:**
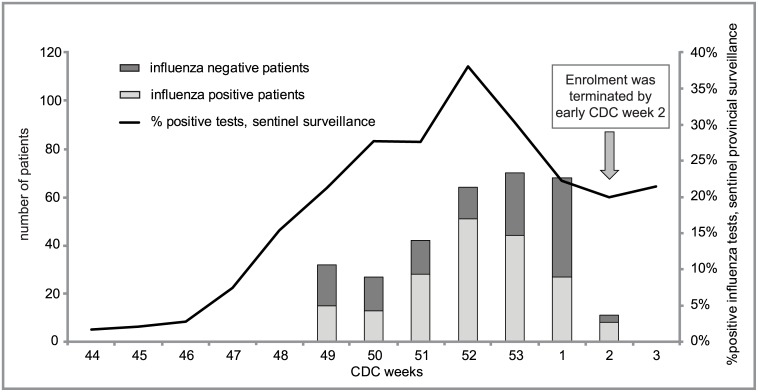
Number of patients included in primary VE analysis by week of hospital admission date, and proportion of positive influenza tests in Quebec sentinel laboratory surveillance system, influenza season 2014/15.

The annual recruitment period for the Quebec sentinel hospital surveillance system, implemented since 2011, spans the peak of the influenza season defined as two consecutive weeks during which at least 15% of weekly samples from the Quebec sentinel laboratory surveillance system test positive for influenza [8]. For the 2014/15 season, this threshold was surpassed beginning in week 48 (November 23–29, 2014) (Fig 1). Systematic respiratory virus surveillance was then conducted among the four acute-care regional hospitals (2 community, 2 academic/tertiary care) serving as sentinel sites and providing care to about 10% of the Quebec population overall.

Only elderly participants with specimen collection within 7 days of illness onset were eligible for inclusion in primary VE analysis. Patients with respiratory symptom onset >72 hours after hospital admission were considered healthcare-associated and were excluded.

Elderly individuals in Quebec are eligible for publicly-funded trivalent influenza vaccine (TIV). Inactivated split or subunit TIV is primarily available for this age group but for the second consecutive season, elderly patients in LTCFs received an MF-59 adjuvanted subunit TIV. Per recommendation of the World Health Organization, all 2014/15 TIV retained the same three influenza vaccine antigens as were also used in 2013/14, including the A/Texas/50/2012(H3N2)-like strain [9].

### Laboratory analysis

Nasal specimens were tested at the provincial public health laboratory using the Luminex RVP FAST version-2 assay which detects influenza A and B and 14 other respiratory viruses. Details are presented elsewhere [8]. Influenza A subtypes were confirmed by reverse transcription polymerase chain reaction (RT-PCR) to detect A(H3) and A(H1)pdm2009 subtypes where otherwise non-subtypeable by Luminex [10, 11].

### Statistical Analysis

Comparison of proportions was by χ2 or Fisher’s exact test and for continuous variables was by Wilcoxon and Kruskal–Wallis nonparametric tests.

VE was estimated by test-negative case-control design [12]. Patients diagnosed with laboratory-confirmed influenza A(H3N2) were considered cases and those testing negative for any influenza were controls. VE was defined as (1-odds ratio)X100% for hospitalization with laboratory-confirmed influenza A(H3N2) among vaccinated compared to non-vaccinated patients. Participants who received the 2014/15 TIV ≥2weeks before illness onset were considered vaccinated. Those for whom vaccination timing was unknown or <2weeks before onset were excluded but explored in sensitivity analyses as indicator variables. Multivariable analyses by logistic regression adjusted for age, underlying comorbidity placing individuals at higher risk of influenza-related complications [13], interval between symptom onset and specimen collection (≤4 days, 5–7 days), hospital site, epidemic week based on hospital admission date (49–51, 52, 53 and 1–2), and primary residence (community, LTCF or other institutional/group setting). Sensitivity analyses explored VE by type of residence, comorbidity, specimen collection interval, and re-classification of patients with unknown vaccination status as vaccinated or as unvaccinated.

## Results

**Fig 2:**
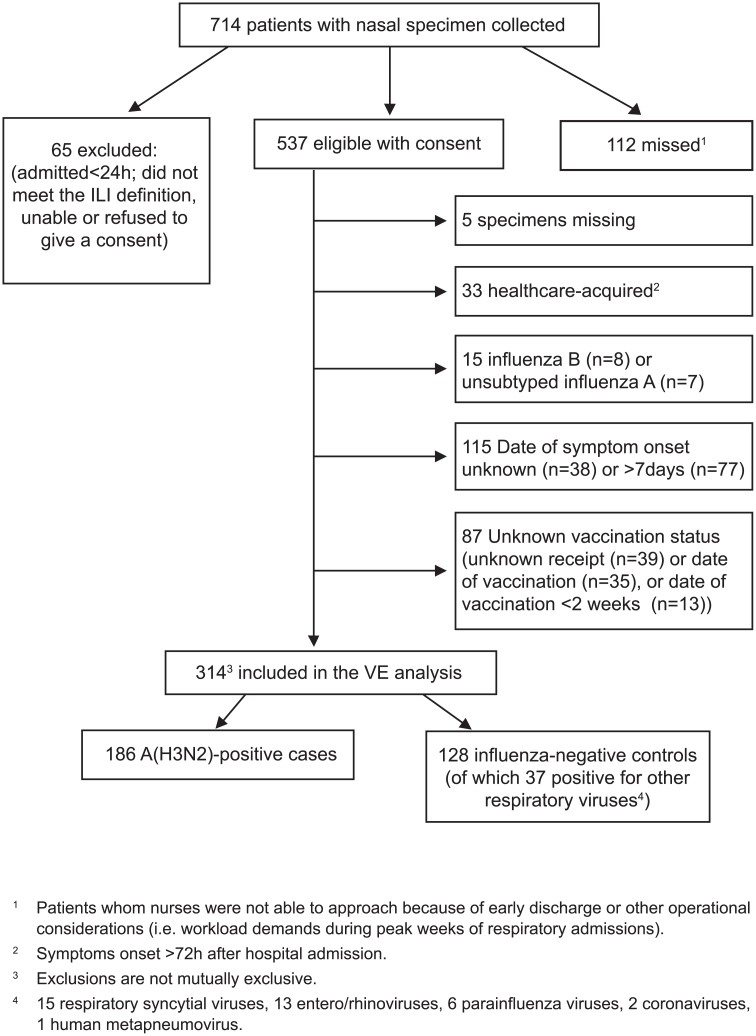
Specimen inclusion/exclusion criteria for primary vaccine effectiveness analysis. ^1^Patients whom nurses were not able to approach because of early discharge or other operational considerations (i.e. workload demands during peak weeks of respiratory admissions); ^2^Symptoms onset >72h after hospital admission; ^3^Exclusions are not mutually exclusive; ^4^15 respiratory syncytial viruses, 13 entero/rhinoviruses, 6 parainfluenza viruses, 2 coronaviruses, 1 human metapneumovirus

During the recruitment period, nasal specimens were collected from 714 elderly patients among whom 537 were eligible for study participation. Further inclusion and exclusion criteria as applied to the current data set for the primary VE analysis are shown in Fig 2 resulting in 314 participants (186 (59%) A(H3N2) cases and 128 (41%) controls) with hospital admission dates spanning from November 30, 2014 (week 49:November 30-December 6, 2014) to January 13, 2015 (week 2:January 11–17, 2015).

The median age of participants was 81.5 years and 30% were ≥ 85years-old (Table 1). Two-thirds were community-dwelling and underlying comorbidity was reported in 91%. The 2014/15 TIV was received by 62% of cases and 59% of controls, the latter comparable to coverage estimates for Quebec elderly overall (62%) [14]. Median age of vaccinated elderly was slightly greater than unvaccinated participants (81.8 vs. 79.0 years; p = 0.0003). A smaller proportion of community-dwelling elderly were vaccinated in 2014/15 compared to those in LTCF (55% vs. 84%; p = 0.002), more comparable to those living in other kinds of institutional/group settings (68%; p = 0.04). Those with underlying comorbidity were more often vaccinated compared to those without (63% vs. 37%; p = 0.008). There was also variation in vaccination coverage by hospital site, lowest in hospital A, located in a region that was affected earliest in the epidemic. Virtually all who were vaccinated in 2014/15 reported also receiving TIV at least once in the past (98% overall, 99% of those for whom this was known).

**Table 1:** Characteristics of patients included in primary VE analysis against A(H3N2) hospitalization in elderly participants ≥ 65 years-old, 2014/15 influenza season, Quebec, Canada.

Variables	Distribution by case status, n(%)				Vaccination coverage within strata, n(%) vaccinated*			
	Overall	A(H3N2) cases	Influenza-negative controls	P value	Overall	A(H3N2) cases	Influenza-negative controls	P value
N(%)	314	186(59)	128(41)		191(61)	116(62)	75(59)	
Age group (years)				>0.05				>0.05
65–74	80(25)	46(25)	34(27)		37(46)	20(43)	17(50)	
75–84	139(44)	79(42)	60(47)		87(63)	52(66)	35(58)	
85+	95(30)	61(33)	34(27)		67(71)	44(72)	23(68)	
Median	81.5	81.5	80.5	>0.05	82.5	82.5	81.5	>0.05
(range)	(65–101)	(65–100)	(65–101)		(65–101)	(66–99)	(65–101)	
Hospital				0.03				>0.05
A	69(22)	45(24)	24(19)		20(29)	13(29)	7(29)	
B	165(53)	102(55)	63(49)		115(70)	71(70)	44(70)	
C	63(20)	27(15)	36(28)		41(65)	22(81)	19(53)	
D	17(5)	12(6)	5(4)		15(88)	10(83)	5(100)	
Male sex	146(46)	87(47)	59(46)	>0.05	98(67)	59(68)	39(66)	>0.05
Residence admitted from:				>0.05				>0.05
Community	209(67)	117(63)	92(72)		114(55)	62(53)	52(57)	
Long-term care facility	32(10)	23(12)	9(7)		27(84)	19(83)	8(89)	
Other institutions	73(23)	46(25)	27(21)		50(68)	35(76)	15(56)	
Underlying comorbidity	287(91)	165(89)	122(95)	0.04	181(63)	109(66)	72(59)	>0.05
Interval from illness onset to specimen collection								
≤4 days	237(75)	141(76)	96(75)	>0.05	146(62)	86(61)	60(63)	>0.05
5–7 days	77(25)	45(24)	32(25)		45(58)	30(67)	15(47)	
CDC week of hospital admission				<0.0001				<0.0001
Week 49–51	97(31)	53(28)	44(34)		48(49)	23(43)	25(57)	
Week 52	64(20)	51(27)	13(10)		35(55)	31(61)	4(31)	
Week 53	71(23)	47(25)	24(19)		47(66)	34(72)	13(54)	
Week 1–2	82(26)	35(19)	47(37)		61(74)	28(80)	33(70)	
Received 2014–15 influenza vaccine*	191(61)	116(62)	75(59)	>0.05	NA	NA	NA	
Any previous seasonal influenza vaccine:				>0.05				>0.05
Yes	215(68)	129(69)	86(67)		187(87)	115(89)	72(84)	
No	87(28)	51(27)	36(28)		1(1)	0(0)	1(3)	
Unknown	12(4)	6(3)	6(5)		3(25)	1(17)	2(33)	
Admitted to intensive care	15(5)	10(5)	5(4)	>0.05	11(73)	8(80)	3(60)	>0.05
Death during hospitalisation	17(5)	12(6)	5(4)	>0.05	13(76)	10(83)	3(60)	>0.05

*≥2 weeks prior to illness onset

NA: not applicable

Fewer cases than controls had underlying comorbidity (89% vs. 95%;p = 0.04) and cases were hospitalized earlier in the epidemic. Conversely, vaccinated participants were hospitalized later in the epidemic (Table 1). A greater proportion of cases than controls were vaccinated among those admitted to ICU (80% vs. 60%) or dying during their hospital stay (83% vs. 60%), but sample size was small and differences were not statistically significant (p>0.05).

Crude VE against elderly A(H3N2) hospitalization was -17% (95%CI: -86 to 26%). When adjusted for age and comorbidity, VE was -23% (95%CI: -99% to 23%) and with full adjustment for recognized confounders was -39% (95%CI: -142% to 20%)(Table 2). In sensitivity analyses, fully-adjusted VE point estimates were improved toward the null with restriction to participants admitted to hospital from the community (-2%; 95%CI: -105 to 49%) or with specimen collection ≤4 days since illness onset (-8%; 95%CI: -104 to 43%). Conversely, the fully-adjusted VE point estimate varied further away from the null with restriction to participants with underlying comorbidity (-51%; 95%CI: -169 to 15%). However, in all analyses confidence intervals spanned the null and were wider as expected with inclusion of more covariates and with subset restriction.

**Table 2:** Estimates of vaccine effectiveness (VE) against influenza A(H3N2)-confirmed hospitalization in elderly participants ≥65 years-old, 2014/15 influenza season.

	VE, % (95% CI)	Number of cases(vaccinated)/number of controls(vaccinated)
**Primary analysis**		
Crude (unadjusted)	-17(-86-26)	186(116)/128(75)
Adjusted for:		
Age (65–74yrs, 75–84yrs, 85+yrs)	-14(-82-29)	
Underlying comorbidity (yes/no)	-27(-103-21)	
Interval from illness onset to specimen collection (≤4/5–7 days)	-17(-85-26)	
Calendar time (weeks 49–51, 52, 53, 1–2)	-35(-120-18))	
Residence	-8(-72-33)	
Hospital site (A-D)	-27(-110-23)	
Adjusted for age and high-risk condition	-23(-99-23)	
Adjusted for all of the above	-39(-142-20)	
**Sensitivity analysis**		
**Restricted to:**		
Admitted from community residence		
Unadjusted	13(-50-50)	117(62)/92(52)
Fully adjusted	-2(-105-49)	
Interval from illness onset to specimen collection ≤4days		
Unadjusted	6(-60-45)	141(86)/96(60)
Fully adjusted	-8(-104-43)	
Underlying comorbidity		
Unadjusted	-35(-119-17)	165(109)/122(72)
Fully adjusted	-51(-169-15)	
**Unknown delay from symptom onset to specimen collection and delay>7days included**		
Unadjusted	-9(-63-27)	227(140)/176(105)
Fully adjusted	-25(-102-23)	
**Patients with unknown vaccinations status classified as vaccinated**		
Unadjusted	-18(-83-24)	223(153)/151(98)
Fully adjusted	-22(-103-26)	
**Patients with unknown vaccinations status classified as unvaccinated**		
Unadjusted	-10(-66-27)	223(116)/151(75)
Fully adjusted	-48(-142-10)	

## Discussion

This study corroborates earlier outpatient and inpatient findings from Canada showing that the 2014/15 influenza vaccine provided little or no protection against the dominant but vaccine-mismatched A(H3N2) strain. Here, we report no vaccine protection against the serious outcome of hospitalization with the 2014/15 antigenically-distinct A(H3N2) epidemic strain among elderly citizens of Quebec, Canada.

Our VE point estimates against elderly A(H3N2) hospitalization for the 2014/15 season, whether partially-adjusted for age and comorbidity alone (-23%; 95%CI: -99% to 23%) or for a fuller range of potential confounders (-39%; 95%CI: -142 to 20%) are similar (within 10%) of the partially-adjusted VE estimate reported by the CIRN hospital-based network (-33%; 95%CI: -104 to 13%)[7]. These VE estimates are substantially lower than CIRN estimates for non-elderly adults for 2014/15 (8%; 95%CI: -102 to 58%). They are also lower than CIRN estimates for the elderly for prior seasons including their mid-season 2013/14 VE against A(H1N1)pdm09-related hospitalization (63%; 95%CI: 35 to 79%) [15] or as reported by CIRN in conference proceedings for the 2012/13 season also against vaccine-mismatched A(H3N2) hospitalization (crude VE = 29%; 95%CI: 18 to 39%) [16]. Our 2014/15 VE estimates are also lower than mid-season VE estimates against outpatient medical visits reported among younger adults by Canada’s SPSN (2%; 95%CI: -49% to 36%)[2], by the United States (US) (12%; 95%CI: -26 to 39%)[17] or by the United Kingdom overall (-2%; 95%CI: −56 to 33%)[18]. VE estimates specific to the elderly were not separately reported in any of these outpatient studies, but among participants ≥50 years-old in the US, VE against outpatient A(H3N2) illness was 14% (95%CI: -31 to 43%)[17]. None of these VE estimates are statistically significant and confidence intervals broadly overlap so that it is not possible to conclude whether VE in hospitalized elderly patients is lower than outpatient VE estimates for elderly or non-elderly adults. Taken together, however, these results challenge assertions [19, 20] that vaccine provides better protection against severe complications than against infection per se, particularly during vaccine-mismatched seasons. In fact, even in young adults in Canada, point estimates of VE against influenza-confirmed hospitalizations reported by CIRN have been consistently lower than estimates against ambulatory illness published by the SPSN each season since 2011 [2, 15, 16, 21].

Consistent findings of negative VE point estimates in relation to hospitalization outcomes in the elderly during the 2014/15 season require some explanation. Confidence intervals around these negative point estimates are wide and cross the null, but broach statistical significance in some analyses. Chance statistical variation, methodological bias, or a true biological phenomenon are among possible explanations. With vaccine coverage of 60%, our sample of just over 300 participants, approximately equally cases and controls, would have been sufficient to detect a statistically-significant VE of at least 50% (in either direction of the null), with 80% power. Sample size requirements increase dramatically as VE more closely approaches zero, as illustrated also by CIRN’s failure to achieve significance despite more than triple the sample size [7]. In that regard negative VE estimates may reflect statistical variation around a true null effect although we cannot rule out that with additional sample size, VE estimates may have crossed toward statistically-significant negative VE. Our study was predicated on the test-negative design, and, as for all observational studies, residual bias and confounding cannot be ruled out. With adjustment for recognized confounders VE estimates were generally reduced but showed greater variability, likely owing to greater sample size requirements to support multiple covariates. It is reassuring, however, that VE estimates were improved toward the null with restriction to more uniform and majority subgroups of participants including those with primary residence in the community, comprising two-thirds of our data set, or among participants with specimen collection ≤4 days since illness onset, comprising three-quarters of participants. VE was reduced among patients with comorbidity, comprising >90% of participants but including a wide variety of conditions, in whom bias related to propensity to vaccinate or to hospitalize likely varies a lot. Factors confounding the association between vaccination and hospitalization risk are more complex than for outpatient medical visits, and this requires more in-depth evaluation generally in the interpretation of VE estimates for hospitalization outcomes, especially for seniors.

Although statistically most consistent with a null vaccine effect overall, it is also prudent to consider whether negative point estimates of VE in the elderly may reflect a true epidemiological finding. In subgroup analysis for the current 2014/15 season, Canada’s SPSN also reported VE estimates against medically-attended A(H3N2) illness that were reduced and slightly negative in patients vaccinated in both 2014/15 and 2013/14 (-15%; 95%CI: -67% to 21%) but positive (i.e. protective) in participants who had received only the 2014/15 vaccine (43%; 95%CI: -29% to 75%)[2]. SPSN findings in repeat vaccine recipients for the 2014/15 season were also associated with wide and overlapping confidence intervals, consistent also with null vaccine effects in both subgroups. A number of other recent studies in Canada, the United States, and Europe have also reported interference from prior receipt of seasonal influenza vaccine [22–26]. While these other studies showed negative interference that sometimes reduces protection (i.e. a lower but still positive VE point estimate), a negative VE estimate would suggest that vaccine interference may sometimes also be associated with increased disease risk. For several decades, the elderly have been a highly and recurrently immunized group, and virtually all of the vaccinated elderly in our study had received TIV in the past. We were not able to stratify by current and/or prior vaccination history and neither has CIRN explored these possible influences, which require further evaluation.

The most noteworthy precedent of negative VE arose during the spring-summer of 2009, and warrants mention here to highlight differences from the current context. Prior receipt of 2008/09 seasonal vaccine was associated with negative VE against the markedly mismatched 2009 pandemic A(H1N1) virus, observed predominantly in non-elderly individuals. This observation was reported by Canada’s SPSN and at least four other studies in Canada [27], subsequently also from Hong Kong [28], the US [29] and Japan [30] and thereafter also in a randomized ferret trial [31]. The Canadian studies showed statistically significant two-fold increased risk (VE of -100%) for medically-attended outpatient A(H1N1)pdm09 illness but risk was not increased for hospitalization outcomes. This is different from the current season’s finding of lesser magnitude, statistically non-significant and more variable vaccine effects (VE of -39%) against A(H3N2) hospitalization in the elderly, for which chance variation around the null and/or methodological considerations may be more likely explanations. Additional studies are needed to definitively resolve the potential concern of vaccine-associated increased risk and/or to clarify the conditions of vaccine mismatch under which it may recur (e.g. antigenic distances [32], immunological cohort effects based on original antigenic prime vs. boost exposures). In fact, the underlying mechanisms and virus-host immunological interactions to explain variability in disease burden and VE by age and influenza subtype require better understanding generally. The reason why elderly people suffer disproportionately from A(H3N2) subtype infections, as per the current season [3, 4] remains a longstanding but unanswered question. Immuno-senescence alone is unlikely to provide the complete explanation since the same exceptional vulnerability is not observed in the elderly in relation to influenza A(H1N1) infection.

Our study has other limitations. A substantial proportion of elderly patients were excluded because they were unable to recall or report important information, such as vaccination status and date of symptom onset. However, their inclusion in indicator sensitivity analyses did not meaningfully alter VE estimates. Vaccine status was self-reported and this may have resulted in exposure misclassification; however, this information was collected prior to influenza diagnosis, minimizing differential misclassification. Studies in other settings, including hospitalized elderly, have reported consistency between self-reported and registry-based influenza vaccine status although that may not directly apply here [33, 34]. We think accuracy of self-reported vaccine status in our study may even be better because information was collected from patients within a shorter period of time since vaccination campaign as compared to the studies cited above. In addition, vaccination coverage in influenza-negative patients enrolled in our study was consistent with that reported from other sources during previous years of the study [8, 14]. Our study was conducted during peak weeks of the influenza season; this may raise concerns about the particular impact of outcome misclassification (i.e. false negative cases) on our VE estimates. We have previously shown that other respiratory viruses remain an important cause of respiratory hospitalization even during peak weeks of the influenza epidemic [8]. The assay we used for influenza diagnosis has been reported elsewhere to have sensitivity >95% (98% for influenza A virus), with comparable proportions testing respiratory virus positive as per individual nucleic acid amplification testing across age groups [35]. Even with sensitivity for influenza detection as low as 70%, in the context of near-perfect specificity, outcome misclassification has been shown to have negligible impact on VE estimates [36]. Although test sensitivity may be lower in elderly adults, it is unlikely to drop below 70%. Finally, in the test-negative study design, when patients with influenza are not censored and can also contribute as controls during another respiratory illness episode, the odds ratio directly estimates the relative risk and is not affected by the rare disease assumption [12].

In conclusion, we report negative point estimates that are statistically non-significant for VE against A(H3N2) hospitalization in the elderly for the 2014/15 season. Our findings are consistent with other outpatient and inpatient studies from Canada, indicating little or, as here, no vaccine protection against the dominant but vaccine-mismatched A(H3N2) epidemic strain. These findings reinforce the need for adjunct measures to protect high-risk individuals, including the elderly, from serious influenza outcomes during vaccine-mismatched seasons and for improved vaccine options over the long-term.
